# Adherence to guidelines of antibiotic prophylactic use in surgery: a prospective cohort study in North West Bank, Palestine

**DOI:** 10.1186/1471-2482-14-69

**Published:** 2014-09-09

**Authors:** Samar MJ Musmar, Hiba Ba`ba, Ala` Owais

**Affiliations:** 1 Department of Community and Family Medicine, Faculty of Medicine and Health Sciences, An-Najah National University, P.O Box 7, 707, Nablus, Palestine; 2 Department of Clinical Pharmacy, Faculty of Medicine and Health Sciences, An-Najah National University, P.O Box 7, 707, Nablus, Palestine

**Keywords:** Surgical prophylaxis, Guidelines, Adherence, Palestine

## Abstract

**Background:**

Surgical site infection is a major contributor to increased mortality and health care costs globally which can be reduced by appropriate antibiotic prophylactic use. In Palestine, there is no published data about preoperative antibiotic use. This study aims to find the pattern of antimicrobial prophylaxis use by evaluating time of the first dose, antibiotic selection and duration after surgery in three governmental hospitals in North West Bank/ Palestine during 2011.

**Methods:**

After approval of Institutional Review Board, a prospective cohort study included a total of 400 abdominal, orthopedic, and gynecological operations which were performed during study period. Trained clinical pharmacists observed selected 301 operations and followed the patient’s files for the three intended study parameters. Compliance of prophylactic antibiotic administration was evaluated according to published guidelines of the American Society for Hospital Pharmacist. Data were analyzed using SPSS version 16 applying descriptive methods. Relationship between guideline compliance and selected operation factors such as type of surgery, patient care unit, and hospital shift, in addition to provider’s age, gender, experience, and specialization were examined applying chi square test. The statistically significant factors with *p* < 0.01 were further analyzed using logistic regression model.

**Results:**

Overall compliance for the studied parameters was very low (2%); only 59.8% received their first dose in appropriate time, 18.5% had appropriate antibiotic selection, and 31.8% of patients received antibiotic in appropriate duration. The OBGYN department had much better compliance regarding timing and duration of antibiotic use (*P* < 0.001), however the proper antibiotic selection was best adhered to for the abdominal surgeries (OR = 3.64, *P* = 0.002). Male providers were statistically significantly much less adherent to the timing of antibiotic dose (OR = 0.28, *p* < 0.001), but better adherent in antibiotic selection (OR = 0.191, *p* = 0.028). Anesthetic technicians showed higher compliance than nurses in timing and duration of antibiotic use.

**Conclusions:**

Lack of guidelines explains the low adherence to appropriate surgical antibiotic prophylaxis in Palestine, with high rate of broad spectrum antibiotic use, long duration and inappropriate time of first dose .We recommend adopting guidelines for prophylaxis and training all health care providers accordingly.

## Background

Surgical site infection (SSI) is an infection that occurs somewhere in the operative field following a surgical intervention. According to Centers for Disease Control and Prevention (CDC), SSI includes incisional and organ space infections [1]. SSI is a major contributor for increased mortality and health care costs [2]. Of nearly 30 million operations in the United States each year, more than 2% are complicated by SSI, mortality rates are 2-3 times higher in patients in whom SSI develops compared with un-infected patients [3].

The risk of SSI depends on patient-related factors such as age, nutritional status and existing infections in addition to surgical factors, such as duration of procedure and the type of operation (clean, clean- contaminated, contaminated, or dirty-infected) [4,5]. The basic principle of antimicrobial prophylaxis in surgery is to achieve adequate serum and tissue drug levels, for the duration of the operation [6].

SSI prevention is important and is based on a combination of preoperative preparation, surgical techniques, peri operative antibiotic prophylaxis and postoperative wound care [7]. There is evidence that appropriate use of antibiotic in surgery is effective in decreasing mortality and health care costs associated with infections developed after surgery [8,9].

In Palestine, there is no published data about antibiotic use in surgery till the time we started our research. Availability of protocols that illustrate antibiotic use in surgery in the hospitals, and the adherence to these protocols are very important items that need evaluation. This study aims to find the pattern of antimicrobial prophylaxis use by evaluating time of the first dose, antibiotic selection and duration after surgery for patients undergoing abdominal, orthopedic and gynecologic operations in three governmental hospitals in North Palestine during 2011.

## Methods

### Study design and setting

This observational non interventional prospective study was performed in the largest three governmental (general) hospitals from January 15 through December 30, 2011. These hospitals are located in the main cities of North West Bank Palestine with a capacity of 213, 105, and 127 beds; all provide orthopedic, general surgery, and Obstetrics &Gynecology (OBGYN) services to the general public. Surgical prophylaxis in the three hospitals is practiced according to general non written guidelines and individual judgment; antibiotics are usually administered by either a nurse (in the ward) or anesthetic technician (in the operating room).

Convenient sampling of all emergent and elective operations in these hospitals meeting inclusion criteria was studied. Institutional Review Board (IRB) of An-Najah National University approval in addition to the approval of the General Directorate of Government Hospitals in the Palestinian MoH in the West Bank was obtained to observe peri operative antibiotic use. Following this approval the General Directors of the selected hospitals (Rafidia surgical hospital in Nablus, Thabet Thabet governmental Hospital in Tulkarem, and Jenin Governmental Hospital in Jenin) also approved the study to be performed in their hospitals. The ethics committee (IRB) waived a formal informed consent for this type of study since it is considered a type of quality assurance whose goal is the improvement of care at the institution.

### Patient population

All patients undergoing abdominal, orthopedic, or gynecologic surgical intervention during the study period were chosen to be our study population. Elective and emergent procedures were included to allow for a comparison between the two types of procedures. The researchers followed the CDC wound classification in order to include only the clean (mainly closed uninfected wound) and clean-contaminated (mainly surgeries entered under controlled conditions and without unusual contamination) [10]. All contaminated or dirty category surgeries in addition to those patients who received therapeutic antibiotic before surgery or those with signs and symptoms of infection after surgery were excluded from the study in order avoid difficulties in distinguishing prolonged prophylaxis from postoperative infection treatment. A total of 400 operations (135 from Hospital 1, 135 from Hospital 2 and 130 from Hospital 3) were studied; 216 (54%) were elective and 184 (46%) were emergent.

### Study variables

Compliance of prophylactic antibiotic administration was evaluated based on the published guidelines of the American Society for Hospital Pharmacist (ASHP) [11]. The following 3 aspects of antimicrobial prophylaxis were assessed:

1- Time of first dose antibiotic: Antibiotic should be administered within 1 hr before incision to achieve prophylactic level during surgery and optimize efficacy. For vancomycin, the infusion should begin within two hours before incision. Doses should be repeated intra operatively if the operation is still in progress two half lives after the first dose.

2- Duration: Antibiotic administration should be discontinued within 24 hours after the end of surgery, to prevent emergence of resistance.

3- Antibiotic selection: In general, inexpensive, non-toxic, and limited-spectrum antibiotic should be used; therefore IV Cefazolin is recommended for most of procedures (orthopedic, gastroduodenal, biliary tract, cesarean section after umbilical cord clamp, and hysterectomy procedures). Cefoxitin is recommended for appendectomy and colorectal procedures; Vancomycin is reserved for patients with beta-lactam allergy.

Each observed operation in the study was classified as adherent or non adherent to each of the three mentioned aspects of compliance.

Patient studied variables were: type of surgery (elective vs emergent),patient care unit (general, orthopedic or OBGYN), and hospital shift (A:8 am -3 pm,B:3-11 pm,C:11 pm-8 am). Health provider variables were age, gender, specialization of provider administering antibiotics (nurse vs anesthetic tech), and years of experience.

### Data collection

Data were collected by two trained clinical pharmacist researchers through frequent visits to the three research sites. Charts of 400 operations which met study criteria were reviewed to collect relevant patient data and compliance data; in addition out of the 400 cases, the researchers were able to observe 301 operations which were carefully selected to represent the three hospitals’ patient care units and shifts. The observation aimed to document incision time, antibiotic first dose time, in addition to health provider characteristics. Data regarding antibiotic selection and post operative antibiotic administration was obtained through file review.

### Data analysis

All analyses were performed with Statistical Package for Social Sciences (SPSS) version 16 statistical program. Descriptive analysis was used to evaluate performance and demonstrate the characteristics of the study sample. Frequency of operations with appropriate first dose time, appropriate type, and appropriate duration were evaluated.

Chi-square test was used to examine the relationship between antibiotic administration and factors such as provider’s age, gender, specialization, years of experience, hospital site, patient care unit, hospital shift, and type of surgery; the results were considered statistically significant at *P* value ≤ 0.05.

To eliminate confounding factors, multivariate analysis was then applied by building a model of independent variables which were significant in univariate analysis at *p* < 0.01. Therefore patient care unit, provider’s age and specialization were entered in each of three logistic regression models for time of antibiotic use, proper antibiotic choice and duration of postoperative antibiotic use.

## Results

Table 1 describes the pattern of antibiotic prophylactic use and types of surgeries included in the study; all operations included in the study received preoperative prophylactic antibiotic and most of them received postoperative antibiotic for 24 hours or more. The duration of the surgeries observed was between 30 minutes to two hours and therefore none of these surgeries needed operative antibiotic redosing.

**Table 1:** Surgical operation types and pattern of antibiotic prophylactic use in the study

**Name of operation**	**Number (%)**	**Type**	**Antibiotic administration**	**Duration of operation**		**Duration of post operative antibiotic**	
				**Mean**	**Range**	**Mean**	**Range**
**OBGYN**	**138 (34.5)**						
Cesarean section	118 (29.5)	Clean	Yes	45 min	40-60 min	24 hr	18-72 hrs
Hysterectomy	15 (3.8)	Clean	Yes	1 hr	50-65 min	20 hr	18-72 hrs
Dilation and curettage	5 (1.2)	Clean	Yes	30 min	20-35 min	Only one dose	---
**General surgery**	**143 (35.8)**						
Laparoscopic cholecystectomy	60 (15)	Clean	Yes	1 hr	50-70 min	24 hr	18-36 hrs
Open cholecystectomy	20 (5)	Clean	Yes	1 hr	50-70 min	48 hrs	24-72 hrs
Appendectomy	63 (15.8)	Clean	Yes	1 hr	50-66 min	36 hrs	24-48 hrs
**Orthopedics**	**119 (29.7)**						
Total hip replacement surgery	51 (12.7)	Clean	Yes	2 hr	90-120 min	4 days	2-6 days
Total knee replacement surgery	54 (13.5)	Clean	Yes	2 hr	90-130 min	3 days	2-5 days
Repair of ankle fracture	4 (1)	Clean-contaminated	Yes	2 hr	90-130 min	2 days	1-3 days
Repair of trochanteric fracture	3 (0.8)	Clean-contaminated	Yes	2 hr	90-130 min	3 days	2-4 days
Repair of femoral shaft fracture	2 (0.5)	Clean-contaminated	Yes	2 hr	90-130 min	3 days	2-3 days
Repair of radius fracture	5 (1.2)	Clean-contaminated	Yes	2 hr	90-125 min	2 days	2-3 days

Table 2 summarizes the characteristics of the studied operations; as expected more than half of operations were done during shift A and were elective surgeries. The health care provider category was observed in 301 operations; mean age for providers was 30.8 (5.4 SD), were mainly (64.5%) anesthetic technicians, with majority (75%) having less than 5 years’ experience.

**Table 2:** Characteristic of the health care facilities and providers for the study sample

**Patient care unit**	**All procedures n = 400**	**Observed procedures n = 301**
	**n (%)**	**n (%)**
Orthopedic surgeries	119 (29.7)	89 (29.6)
Abdominal surgeries	143 (35.8)	107 (35.5)
OBGYN surgeries	138 (29.5)	105 (34.9)
**Shift time**		
A (8 am – 3 pm )	233(58.2)	175(58.1)
B (3 pm – 11 pm)	106(26.5)	80(26.6)
C (11 pm – 8 am )	61(15.2)	46(15.3)
**Operation type**		
Elective	216 (54)	164 (54.4)
Emergent	184 (46)	137 (45.6)
**Provider specialty**		
Practical nurse		107 (35.5)
Anesthetic technician		194 (64.5)
**Provider age**		
Less than 30		226 (75.1)
30-40		65 (21.6)
More than 40		10 (3.3)
**Provider gender**		
Male		149 (49.5)
Female		152 (50.5)
**Provider experience (year)**		
1-5		226 (75.1)
6-10		20 (6.6)
More than 10		55 (18.3)

Among 301 patients undergoing abdominal, orthopedic, and gynecological procedures, only 59.8% received their first dose with appropriate time (Table 3). Antibiotic selection for all 400 studied procedures was consistent with published guidelines for only 18.5%, and was discontinued within 24 hours post operation for only 31.8% of patients.

**Table 3:** **Compliance**^**ɤ **^**with antibiotic prophylaxis in the 3 research sites**

**Variable**	Adherence in dosing time n = 180* n (%)	**P value**	Adherence in antibiotic selection n = 74** n (%)	**P value**	Adherence in duration of antibiotic use n = 127** n (%)	**P value**
**Hospital**		**0.023**		**0.21**		**0.27**
Hospital 1	72 (69.9)		30 (22.2)		49 (36.6)	
Hospital 2	55 (53.9)		19 (14.1)		37 (27.4)	
Hospital 3	53 (55.2)		25 (19.1)		41 (31.5)	
**Patient care department**		**<0.001**		**<0.001**		**<0.001**
Orthopedic	42 (47.1)		12 (10.1)		24 (20.2)	
Abdominal	55 (51.4)		53 (37.1)		33 (23.2)	
OBGYN	83 (79.1)		9 (6.5)		70 (50.7)	
**Hospital shift time**		**0.26**		**0.43**		**0.87**
A(8 am – 3 pm)	109 (62.3)		40 (17.2)		76 (32.8)	
B(3 pm – 11 pm)	42 (52.5)		24 (22.6)		33 (31.1)	
C(11 pm – 8 am )	29 (63.1)		10 (16.4)		18 (29.5)	
**Type of surgery**		**0.24**		**0.04**		**0.61**
Emergent	77 (56.2)		42 (22.8)		61 (33.2)	
Elective	103 (62.8)		32 (14.8)		66 (30.7)	

^ɤ^altogether compliance of all three was only in six procedures (2%).

*Out of observed procedures (301).

**Out of all studied procedures (400).

Of all studied and observed procedures only 6 (2%) were compliant with surgical prophylaxis studied guidelines altogether (dosing time, antibiotic choices, and postoperative duration of antibiotic use); these six procedures were from the three hospitals and were done in all shifts. When we compared between the three research sites (Table 3), there was no significant statistical difference except for the timing for first dose (*p* = 0.023). Tables 3 and 4 show that OBGYN department had a much better compliance regarding timing compared to orthopedics (OR =0.15, CI: 0.06-0.38,*p* <0.001) and abdominal procedures (OR = 0.25,CI: 0.10-0.6,*p* = 0.003). The duration of antibiotic use was also found to be adhered to in OBGYN department much more than orthopedics (OR = 0.27, CI: 0.13-0.54, *p* < 0.001), and abdominal procedures (OR = 0.39, CI: 0.20-0.77, *p* = 0.007), however abdominal procedures were more likely to adhere to proper antibiotic choice (OR = 3.64. CI: 1.57-8.41, *p* = 0.028).

**Table 4:** Compliance with antibiotic prophylaxis according to health care facilities and provider characteristics using multivariate analysis

**Variable**	**Adherence in dosing time OR (CI)**	**P value**	**Adherence in antibiotic selection OR (CI)**	**P value**	**Adherence in duration of antibiotic use OR (CI)**	**P value**
**PCU**						
Orthopedic	0.15 (0.06-0.38)	<0.001	0.67 (0.25-0.17)	0.378	0.27 (0.13-0.54)	<0.001
Abdominal	0.25 (0.10-0.63)	0.003	3.64 (1.57-8.41)	0.378	0.39 (0.20-0.77)	0.007
OBGYN*	1		1		1	
**Gender**						
Male	0.28 (0.16-0.48)	<0.001	1.19 (1.07-3.41)	0.028	0.79 (0.46-1.34)	0.385
Female*	1		1		1	
**Specialization**						
Nurse	0.24 (0.13-0.43)	<0.001	1.67 (0.91-3.08)	0.095	0.44 (0.23-0.83)	0.012
Anesthesia* technician	1		1		1	

*Reference category.

*OR*: Odds Ratio.

*CI*: Confidence Interval.

Our study findings in Tables 4 and 5 show that health provider who administers the antibiotic prophylaxis may influence adherence; for example male providers were statistically significantly more adherent to the antibiotic choice (OR = 1.19,CI:1.07-3,41, *p* = 0.028), but much less adherent in timing for first dose (OR = 0.28, CI: 0.16-0.48, *p* < 0.001). Interestingly the anesthetic technicians showed a higher compliance than nurses in timing (OR = 0.24, CI: 0.13-0.43, *p* <0.001) and duration (OR = 0.44, CI: 0.23-0.83, *p* =0.012) of antibiotic use.

**Table 5:** Compliance with antibiotic prophylaxis according to health provider characteristics

**Variable**	**Adherence in dosing time n = 180 n (%)**	**P value**	**Adherence in antibiotic selection n = 74 n (%)**	**P value**	**Adherence in duration of antibiotic use n = 88 n (%)**	**P value**
**Age**		**0.81**		**0.16**		**0.38**
Less than 30	136 (60.2)		51 (22.6)		69 (30.7)	
More than 30	44 (58.7)		23 (30.7)		19 (25.3)	
Total	180 (59.8)		74 (24.6)		88 (29.3)	
**Gender**		**<0.001**		**0.013**		**0.23**
Male	70 (47.0)		46 (30.9)		39 (26.2)	
Female	110 (72.4)		28 (18.4)		49 (32.5)	
Total	180 (59.8)		74 (24.6)		88 (29.3)	
**Specialization**		**<0.001**		**0.005**		**<0.001**
Nurse	39 (36.4)		36 (33.6)		17 (15.9)	
Anesthetic technician	141 (72.7)		38 (19.6)		71 (36.8)	
Total	180 (59.8)		74 (24.6)		88 (29.3)	
**Years of experience**		**0.81**		**0.16**		**0.38**
Less than 5	136 (60.2)		51 (22.6)		69 (30.7)	
More than 5	44 (58.7)		23 (30.7)		19 (25.3)	
Total	180 (59.8)		74 (24.6)		88 (29.3)	

## Discussion

The most important finding in this study is the absence of any written agreed upon guidelines for antibiotic surgical prophylaxis in all governmental hospital sites studied. This finding explains the very low adherence to international guidelines found in the study (only six observed ones). Low adherence is shared by other studies in the region; For example the Jordanian study found that none of the observed cardiac operations was adherent to all antimicrobial prophylaxis guidelines with wide variation in adherence to selected parameters studied [12]. The Iranian study also found only one surgical procedure of the observed 155 to be adherent to all parameters of prophylaxis guidelines with varying degrees of compliance in different parameters [13], and the Turkish study found only 13.7% of the perioperative antibiotic prophylaxis given were appropriate and correct [9].

Even in USA where following surgical prophylaxis guidelines is an expected practice, a study of medicare in patients undergoing different kinds of surgical procedures demonstrated that 55.7% of patients received their antibiotic dose within one hour before the surgical incision , and antimicrobial prophylaxis was discontinued within 24 hours after surgery for only 40.7% of patients [14].

Only one hospital in our study which is the main site for medical and nursing students’ training had better adherence in the first time dosing of antibiotics, in addition the OBGYN department showed a very high compliance to dosing time. Time of antibiotic administration before surgery is very important issue in prophylaxis and infection prevention, microorganisms are expected to enter body fluids and tissues from the time of incision until the injury is closed, during this time the antimicrobial level must be in the inhibitory level in serum [15].

One of factors associated with poor adherence in time of prophylaxis administration in our study is the administration of antibiotics by nurses on the ward at fixed clock rounds instead of adjusting this to the time before surgery, this is also the cause for inappropriate time documented in a study in a multicenter audit in Dutch hospitals [16]. The timely administration of first dose by anesthesia technician is also shared by American study which found that timely administration improves when antibiotic prophylaxis is given in the operation room [17].

Both selection of antibiotic for prophylaxis (18.5%) and duration of post-operative use (31.8%) were far from adherence to the guidelines in our study. For most types of orthopedic, abdominal and gynecological operations, single pre-operative dose of the first generation cephalosporin is recommended; further post-operative doses are not needed and the antibiotic should be discontinued within 24 hours post operation [18]. However because of lack of protocols, and hospital supply availability of antibiotics, personal judgments of treating physicians may explain the tendency to use broad spectrum or combination antibiotics and to continue use beyond 24 hours in our study. These findings are shared with Jordanian study which found that neither antibiotic choice(1.7%) nor duration(39.4%) were appropriate [12], the Turkish study [9] which also found that prolonged antibiotics prophylaxis was used in 56.9%, however the US study where protocols are usually followed showed excellent compliance (92.6%) in antibiotic selection [14].

The general surgery department in this study showed a better adherence in selection of antibiotic compared with OBGYN and orthopedic department, a finding shared with a Turkish study which showed that general surgeons use antibiotic prophylaxis more appropriately [19]. On the other hand OBGYN had much better adherence to the dosing time and duration possibly because most of surgeries done in this department are cesarean sections which follow agreed on non-written protocol.

An interesting finding is the tendency of female providers to be more adherent to the time of first dose and males to show better adherence to the selection of antibiotic, it is difficult to explain this finding since all providers regardless of their gender receive the same training and role in performing their jobs according to their qualification.

Although there is no written protocol, it seems that the anesthetic technicians were much more compliant to the time of first dose and postoperative antibiotic duration. This is possibly because the technician's main training and job skills are related to operation room; on the other hand nurses have wider scope of work for patients on the ward.

## Conclusion

None of the hospitals studied is following guidelines for perioperative prophylaxis. This explains the low adherence to appropriate surgical antibiotic prophylaxis in Palestine, with high rate of broad spectrum antibiotic use; long duration and inappropriate time of first dose.We recommend adopting guidelines for surgical prophylaxis in addition to the need to train all health care providers accordingly. Role of anesthesia technician in administering prophylactic antibiotic seems to be important and needs to be emphasized.

## Competing interests

The authors declare that they have no competing interests.

## Authors’ contributions

SM participated in the study design and coordination of the study protocol. HB worked on preparing the background and literature review. Both HB and AO performed the field work of data collection in addition to statistical analysis. SM reviewed the statistical analysis and drafted the results and discussion. HB and SM drafted the manuscript and all three authors reviewed and approved it.

## Pre-publication history

The pre-publication history for this paper can be accessed here:

http://www.biomedcentral.com/1471-2482/14/69/prepub
